# Fabrication, characterization, and biological assessment of multilayer laminin γ2 DNA coatings on titanium surfaces

**DOI:** 10.1038/srep23423

**Published:** 2016-03-21

**Authors:** Guoli Yang, Jing Zhang, Wenjing Dong, Li Liu, Jue Shi, Huiming Wang

**Affiliations:** 1 Department of Implantology, Stomatology Hospital, School of Medical, Zhejiang University, Yan’an Road, Hangzhou, P. R. China; 2 Department of Implantology, Stomatology Hospital of Xuzhou, P. R. China; 3 Department of Prosthodontics, Stomatology Hospital, School of Medical, Zhejiang University, Yan’an Road, Hangzhou, P. R. China; 4 Department of Oral and Maxillofacial Surgery, Stomatology Hospital, School of Medical, Zhejiang University, Yan’an Road, Hangzhou, P. R. China

## Abstract

The purpose of this work was to fabricate a multilayer laminin γ2 DNA coating on a titanium surface and evaluate its biological properties. A multilayer laminin γ2 DNA coating was fabricated on titanium using a layer-by-layer assembly technique. The rate of coating degradation was evaluated by detecting the amount of cDNA remaining. Surface analysis using X-ray photoelectron spectroscopy, atomic force microscopy, and surface contact angle measurements revealed the multilayer structure to consist of cationic lipid and confirmed that a laminin γ2 DNA layer could be fabricated on titanium via the layer-by-layer assembly process. The transfection efficiency was highest for five layers in the multilayer structure. HEK293 cells cultured on the multilayer films displayed significantly higher adhesion activity than the control group. The expression of laminin γ2 and the co-localization of integrin β4 and plectin were more obvious in HN4 cells cultured on the multilayer laminin γ2 DNA coating, while weak immunoreactivities were observed in the control group. We concluded that the DNA-loaded multilayer provided a surface with good biocompatibility and that the multilayer laminin γ2 DNA coating might be effective in improving cell adhesion and the formation of hemidesmosomes on titanium surfaces.

Over the past few years, dental implants have become an important prosthetic because of their comfort and appealing aesthetics^1^. The success of a dental implant treatment depends not only on the healing of hard tissues but also on the formation of soft tissues. It has been reported that attachment loss of soft tissues around dental implants is one of the most important causes of implant failure^2^. A good biological seal between the soft tissue and the implant may prevent oral bacteria and their products from penetrating the body and minimize the risk of peri-implantitis. Thus, strengthening the attachment of the epithelium to the implant surface is crucial to the success of dental implants.

Like the junctional epithelium, the peri-implant epithelium makes close contact with the surface of the implant via a unique structure. It has been reported that the peri-implant epithelium attaches to the surface of the implant via hemidesmosomes and a basal-lamina-like extracellular matrix, which is termed the internal basal lamina^3^,^4^,^5^. Atsuta *et al*.^1^ reported that hemidesmosomes and internal basal lamina, which serve as adhesion structures, formed in the apical portion of the implant–peri-implant epithelium interface. However, these adhesion structures have not been observed in the upper-middle portion of the interface^6^,^7^. Ultrastructural observations demonstrate that the internal basal lamina is divided into the lamina densa and the lamina lucida. The lamina densa connects to the implant surface, while the lamina lucida connects to peri-implant epithelium cells; this connection is reinforced by the presence of hemidesmosomes. Several studies have proposed that the internal basal lamina is a simple extracellular matrix without a network structure, in which integrin α6β4 and laminin-5 form a complex to sustain attachment of the peri-implant epithelium and implant surface^4^,^8^.

Laminin-5 is a heterotrimer consisting of α3, β3, and γ2 subunits^9^. A number of studies have demonstrated that laminin-5 is an important component of the internal basal lamina and contributes to cell adhesion associated with integrin α6β4 at hemidesmosomes^10^,^11^. Several investigators have proposed that monomeric laminin-5 molecules with anchoring filaments bridge integrin α6β4 to type VII collagen and provide a significant force that promotes cohesion of the dermis and epidermis^12^. The adhesive role of laminin-5 has also been confirmed by the detection of circulating autoantibodies against epitopes of laminin-5 in patients with acquired blistering skin disorders, which are characterized by dermal–epidermal cleavage^13^. Differing from other laminins, laminin-5 contains a unique γ2 chain^14^. Studies indicate that the γ2 globular domain IV drives deposition of laminin-5 into the extracellular matrix and sustains cell adhesion^12^. A previous study found that the laminin γ2 chain could be secreted as a monomer without any expression of the α3 or β3 chains or as a γ2/β3 heterodimer into culture medium from human cancer cell lines^10^,^15^. In this way, increasing the expression of the laminin γ2 chain can enhance the deposition of laminin-5. This is also beneficial for the formation of biological seal between an implant and the soft tissue.

Currently, the use of therapeutic protein delivery systems is still limited by protein instability and immunogenicity^16^. DNA delivery has become an important technique for the production of recombinant proteins over the past few decades. The exogenous gene can be expressed after a short period of time to produce recombinant proteins by inserting the recombinant gene into the host genome, which requires the use of a vector^17^,^18^. Compared with viral vectors, nonviral vectors are easy to manufacture, less costly, and have a lower toxicity^19^. However, it is necessary to provide spatial and temporal control over the release and delivery of DNA from implant surfaces^20^. The layer-by-layer assembly technique was first introduced by Decher^21^. It is a simple and versatile method of coating biological templates, suitable for various biomedical applications^21^,^22^. It can produce films by creating attractive electrostatic forces. Our previous study demonstrated that gene-functionalized titanium surfaces, constructed via a layer-by-layer approach, can transfer plasmid cDNA into cells^23^.

Laminin-5 is an important protein in the extracellular matrix, and it has been found to be associated with integrin α6β4, with which it forms a transmembrane system in epithelial cells. This system can strengthen attachment of the epithelium and implant surface. This study aims to investigate the effects of the transmembrane system on the cuffs of peri-implant tissues. The purpose of this study was to form laminin γ2 gene coatings onto titanium surfaces using a layer-by-layer self-assembly process and evaluate its characterization. In addition, the biological properties of HEK293 cells attached to gene-coated titanium surfaces and the formation of hemidesmosomes in HN4 cells was investigated.

## Materials and Methods

### Biomaterials

Plasmids, specifically pReceiver-M61C-LAMC2, were constructed by GeneCopoeia (USA) and amplified at the School of Basic Medical Science, Zhejiang University. Chitosan (CS, molecular weight, 100,000) was purchased from Qingdao Yunzhou Biochemistry (China). Fluorescein-isothiocyanate-labeled phalloidin, Triton X-100, CelLytic buffer, and hyaluronic acid (HA) were purchased from Sigma Chemical Co. (MO, USA). A Label IT^®^ TM-Rhodamine labeling kit was purchased from Mirus Bio, LLC. (USA). Alamarblue and Lipofectamine LTX Plus reagent were purchased from Invitrogen (USA). Bovine serum albumin was purchased from Shanghai Sangon Biological Engineering Technology and Services (China). Hoechst 33258 DNA dye was purchased from Beyotime (China). Anti-laminin γ2 mouse monoclonal antibody, anti-integrin β4 mouse monoclonal antibody and anti-plectin rabbit monoclonal antibody were purchased from Abcam (USA). Goat anti-mouse IgG conjugated with Alexa 488 and goat anti-mouse IgG conjugated with Alexa 594 were purchased from Invitrogen (USA). Goat anti-rabbit IgG conjugated with rhodamine was purchased from Jackson ImmunoResearch Laboratories (USA). A human laminin-5 ELISA kit was purchased from R&D Technologies. Flat pure titanium plates 10 × 10 × 1 mm in size were purchased from Zhejiang Guangci Medical Appliance Company (China).

### Surface treatment of titanium disks

Samples were polished until they were similar in shape to transgingival implants. Specifically, they were polished with silicon carbide paper of various grain sizes and washed for 15 min in an ultrasonic cleaner with, sequentially, acetone, 75% alcohol, and distilled water and then dried in a nitrogen atmosphere.

### Fabrication of multilayered DNA coatings

Multilayered gene coatings were generated on smoothed titanium disks using the layer-by-layer technique as described by Liu *et al.*^23^ and Jiang *et al.*^24^ with a few modifications. In general, chitosan was dissolved in 2 vol% acetic acid at a concentration of 5 mg/ml. Hyaluronic acid (HA) was dissolved in distilled water to give a concentration of 0.5 mg/ml. Meanwhile, cationic lipid and cDNA complexes were prepared according to the manufacturer’s protocol. Briefly, 50 μL (1 μg/μL) of plasmid DNA was mixed with 10 mL Opti-MEM (Gibco Life Technologies, Grand Island, NY, USA), 50 μL PlusTM reagent and 100 μL Lipofectamine LTX to form the liposome-DNA complexes (LDc). First, the smoothed titanium disks were immersed in chitosan solution for 30 min, producing a precursor layer with a stable positive charge to initiate the layer-by-layer assembly process. Then the chitosan-coated titanium disks were dipped into the HA solution (0.5 mg/ml) and maintained for 5 min at room temperature to adsorb HA onto the surface electrostatically. The next steps were performed according to the method described by Liu *et al.*^23^. In this method, the number of layers was defined as the number of procedures for the adsorption of both LDc and HA. For example, five layers were associated with adsorption of CS-(HA-LDc)_5_. The CS-(HA-LDc)n was assembled in one, three, five, seven, or nine layers. Similarly, the CS-(HA-Lip)n was assembled from CS, HA and Lipofectamine LTX without DNA.

### Labeled DNA loaded on titanium substrate surface

Plasmid DNA (pDNA) was labeled with rhodamine using a Label IT labeling kit according to the manufacturer’s protocol. The procedure for the layer-by-layer assembly of loading rhodamine-labeled cDNA onto the titanium surface was performed as described but in the dark. Then cDNA was visualized with a Nikon Eclipse 80i fluorescence microscope (Nikon Corp., Melville, NY, USA) with DXM1200F CCD. The fluorescence of the rhodamine-labeled cDNA on the surface of the plate was analyzed over a 1.2 mm^2^ area using Image-Pro version 6.0 software (Media Cybernetics Corp., USA). Results were obtained using a calibration curve, which was prepared by depositing known amounts of plasmid DNA onto the CS and HA-adsorbed titanium substrate surface. CS-(HA-Lip)9 without plasmid was established as a blank control. Three samples from each group were analyzed.

### Degradation of multilayer coatings

To investigate coating degradation, titanium disks with CS-(HA-LDc)n were immersed in phosphate-buffered saline (PBS) buffer (pH = 7.4) at 37 °C. Every 2 days, titanium substrates were taken out and rinsed with deionized water for visualization with a Nikon Eclipse 80i fluorescence microscope (Nikon Corp., Melville, NY, USA) with DXM1200F CCD. The average fluorescence intensity was measured as described. Each group contained three samples.

### Surface characterization analysis

X-ray photoelectron spectroscopy was performed using an AXIS ULTRA^DLD^ spectrometer (Shimadzu, Japan) with a monochromatized Al Kα X-ray source operating at 15 kV and 8 mA. A survey scan (0–1000 eV binding energy range) and a high-resolution scan of carbon, titanium and nitrogen were run for each specimen. Binding energies were calibrated with C1s (284.6 eV). Contact angle measurements were determined using a dynamic contact angle system (SL200B, Solon Tech. Inc. Ltd., Shanghai, China), with ultrapure water as wetting agent, at room temperature. Each contact angle reported here is the mean of at least three independent measurements. Atomic force micrographs were recorded using the tapping mode in air at 20–25 °C using a Nano-scope I multimode scanning force microscope (Digital Instruments, Santa Barbara, CA, USA).

### Cell culture

HEK293 cells were obtained from the American Type Culture Collection (ATCC). Human head and neck squamous cell carcinoma cell line HN4 was acquired from the Surgery Laboratory of the First Affiliated Hospital of Zhejiang University (Zhejiang, China). Both HEK293 and HN4 cells were cultured in DMEM (Gibco, USA) supplemented with 10% fetal bovine serum (Gibco, USA) at 37 °C under 5% CO_2_ atmosphere. Penicillin (100 U/ml) and streptomycin (100 mg/ml) (Invitrogen, Grand Island, NY, USA) were added to the culture medium.

### *In-vitro* cDNA transfection

For transient expression, subconfluent HEK293 cells and HN4 cells were seeded at a density of 2 × 10^4^ cells/well onto the CS-(HA-LDc)5 coating surface in 24-well culture plates. Then, 1 day, 4 days, 7 days, and 9 days later, the medium was gently removed and samples were fixed with 4% paraformaldehyde/PBS at room temperature for 20 min. After washing with PBS, the cells were incubated with rhodamine phalloidin for 30 min, followed by counterstaining with Hoechst 33258 DNA dye for 5 min in the dark. Transfection efficiency was detected using a Nikon Eclipse 80i fluorescence microscope (Nikon Corp., Melville, NY, USA) with DXM1200F CCD. Triplicate samples were used in all cases. The transfection efficiency was calculated using the following equation:


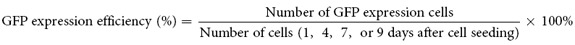


Green fluorescent protein (GFP) expression efficiency was expressed as mean ± standard deviation for three different substrates and was replicated in a separate second run.

### Measurement of attached cell numbers in early stages

To study the cell morphology and attachment of HEK293 cells on CS-(HA-LDc)5-coated surfaces, CS-(HA-Lip)5-coated surfaces, and control surfaces, cells were harvested on the disks at a density of 1 × 10^4^/well in 24-well plates. After 1 h, 6 h, 12 h, and 24 h of incubation, unattached cells were removed with three washes in 0.01 M PBS and the samples were fixed with 4% paraformaldehyde/PBS at 4 °C for 20 minutes. The samples were then washed three times in PBS, followed by nonspecific binding blocked with 0.5% bovine serum albumin for 30 min at room temperature. Actin microfilaments were stained using fluorescein-isothiocyanate-labeled phalloidin at a 1:40 dilution. After washing three times with PBS, nuclei were stained by incubation with Hoechst 33258 DNA dye for 5 min. Finally, cells cultured on titanium disks were observed and photographed with a fluorescence microscope (Eclipse-80i; Nikon, Tokyo, Japan). Cell numbers and cell areas were measured in ten randomly selected areas on each sample using Image-Pro version 6.0 software (Media Cybernetics Corp., USA). Each group contained three disks.

### Cell proliferation and adhesion in later stages

HEK293 cells were seeded on different multilayer-coated titanium films or control surfaces with a density of 1 × 10^4^/well. After 2 days, 4 days and 6 days seeding, unattached cells were removed with three washes in 0.01 M PBS and the number of cells on each titanium disk was determined using alamarblue cell viability reagent. Briefly, at desired time intervals, culture medium was replaced with 500 μl fresh medium and 50 μl of alamarblue was added to each well and incubated at 37 °C for 4 h. The fluorescence intensity of the mixed solution was measured using a SpectraMax microplate reader (Molecular Devices, USA) with excitation and emission wavelengths of 540 and 590 nm, respectively. Each group contained three disks. The mean value served as the final result.

### Quantification of laminin-5

HEK293 cells were seeded on Ti-CS-(HA-LDc)5, Ti-CS-(HA-Lip)5, and uncoated titanium disks at a density of 2 × 10^4^ cells/well. After 2 days, 4 days, and 6 days culture, the medium was removed, the cell layer was rinsed with PBS, and the cells were lysed with CelLytic buffer. The content of laminin-5 was determined using a human laminin-5 ELISA kit, which uses horse-radish-peroxidase-tagged laminin-5 antibodies as the substrate, according to the manufacturer’s instructions. Briefly, a 50 μL volume of testing sample was incubated with 100 μL working assay at 37 °C for 15 min in a 96-well plate. The reaction was then terminated by the addition of 50 μL stop solution to each well. The wells were then read at 450 nm with a spectrophotometer. The standard curve linear regression equation was calculated using the standard concentration and the corresponding optical densities. Then the optical densities and corresponding sample concentration were determined using the sample and the regression equation.

### Immunofluorescence microscopy

HN4 cells were seeded with a density of 2 × 10^4^ cell/well onto the control group surface and the CS-(HA-LDc)5 coating surface in 24-well culture plates. After 48 h, the medium was gently removed and samples fixed with 4% paraformaldehyde/PBS at room temperature for 20 min. After washing with PBS, the cells were incubated overnight with an anti-laminin γ2 mouse monoclonal antibody at 4 °C. After washing with PBS, the cells were incubated with a secondary antibody, goat anti-mouse IgG conjugated with Alexa 594. Actin microfilaments were stained with fluorescein-isothiocyanate-labeled phalloidin and nuclei were stained with Hoechst 33258 DNA dye.

Integrin β4 and plectin are important components of hemidesmosomes in epithelial cells. The co-localization of integrin β4 and plectin was undertaken to detect the structure of the hemidesmosomes. Thus, other samples were incubated overnight with the solution containing anti-integrin β4 mouse monoclonal antibody and anti-plectin rabbit monoclonal antibody at 4 °C. Then the cells were incubated with two secondary antibodies: goat anti-mouse IgG conjugated with Alexa 488 and goat anti-rabbit IgG conjugated with rhodamine. Nuclei were stained by Hoechst 33258 DNA dye. The cells were examined and photographed using a fluorescence microscope under ×400 magnification.

### Statistical analysis

Data were tested for normal distribution and statistically analyzed using one-way analysis of variance (ANOVA). Group means and standard deviations were used to calculate each parameter. The software of IBM SPSS statistics 19.0 (SPSS) was used for all statistical analysis. Significance was considered for *P* < 0.05.

## Results

### Quantity of DNA fabricated on the titanium surface and degradation of multilayered coatings

**Figure 1:**
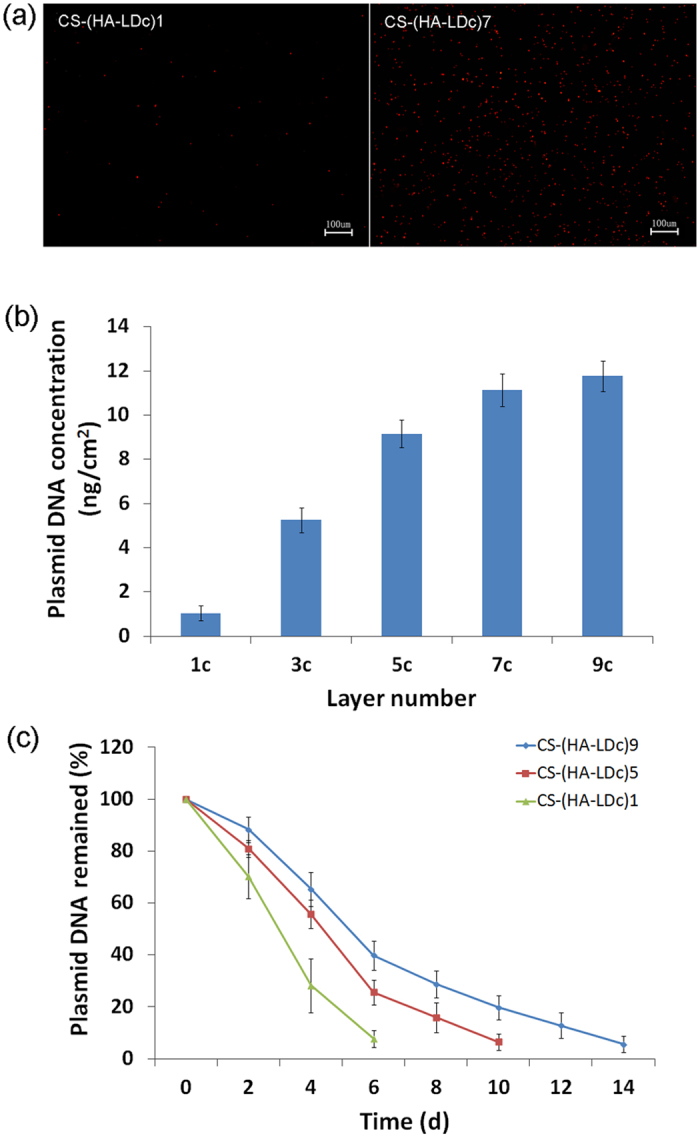
Amount of DNA fabricated on the titanium surface and degradation of multilayered coatings. **(a)** Fluorescence microscope images of labeled CS-(HA-LDc)1 coating and CS-(HA-LDc)7 coating on titanium surface under ×100 magnification. **(b)** Influence of layer number on the amount of loaded plasmids. n = 3 for each group. Data are displayed as mean ± SD. **(c)** DNA remained on porous CS-(HA-LDc)n titanium surface after incubated in PBS buffer for different degradation time. Data shown were obtained from a single experiment (n = 3 for each group), which was performed three times. Data are displayed as mean ± SD.

The coatings were fluorescence-labeled on the titanium disks, as shown in Fig. 1a. During adsorption cycles, the amount of DNA loaded onto the titanium increased. As shown in Fig. 1b, the density of plasmids reached 5.256 ± 0.557 ng/cm^2^ on the CS-(HA-LDc)3 surface and 11.128 ± 0.732 ng/cm^2^ on the CS-(HA-LDc)7 surface. In Fig. 1c, it shows the content of plasmids remaining on titanium substrates with one, five, and nine bilayers of coating at different time intervals. Results have shown that the greater the number of assembly layers, the longer the period over which the plasmids are released. The rate of plasmid release was fast in the first 6 days and then became slow.

### X-ray photoelectron spectroscopy

**Figure 2:**
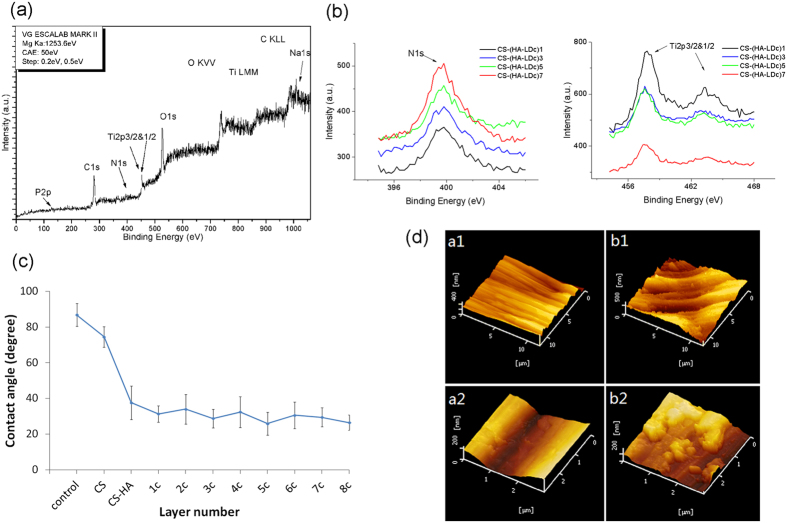
Surface characterization analysis of the coatings on titanium. **(a)** XPS spectra of titanium surface coated with CS-(HA-LDc)3 coating. **(b)** XPS N 1s spectra of CS-(HA-LDc)n (left) and Ti 2p3/2&1/2 spectra of CS-(HA-LDc)n (right). **(c)** Contact angles of the titanium surface with different deposition cycles. **(d)** AFM images of uncoated titanium surface and Ti discs with coatings. (a1) (a2) control, (b1)(b2) CS-(HA-LDc)5, a2 and b2 show the high-resolution image.

X-ray photoelectron spectroscopy was conducted to determine the adsorption of polyelectrolytes on the coated titanium surface. As shown in Fig. 2a, there are Ti, O, C, N, Na, and P signals on the CS-(HA-LDc)3 surface. For all surfaces, a double peak with broad shoulders was observed at about 457.6 and 463.3 eV, which indicated that TiO_2_ was the main form of titanium on the samples. The N1s signal appeared at about 399.8 eV. As the number of layers increased, the signal intensity of N was strengthened and the Ti signal was weakened (Fig. 2b). The increase in the N signal indicated the adsorption of HA (Na, N) and DNA (N, P). However, the signal for P was weaker than that for other elements.

### Contact angle measurement

Contact angle measurement can reflect the wettable properties of the measured substrates. As shown in Fig. 2c, the contact angle of uncoated titanium surface was 88°, but decreased to 74.51° on titanium assembled with chitosan. Hyaluronic acid is more hydrophilic than chitosan. The contact angle decreased and fluctuated between 35° and 25° when HA and LDc were contained in the outmost layer. As a result, the wettability of multilayered titanium films differed from that of native titanium surfaces; this suggests that the CS-(HA-LDc)n coatings had been successfully constructed using the layer-by-layer technique. This change in the wettability of titanium may be attributable to the hydrophilic nature of HA and LDc.

### Morphological characterization

The surface topography of a titanium surface coated with CS-(HA-LDc)5 and an uncoated control titanium surface were observed (Fig. 2d). The uncoated specimen showed parallel grooves on the titanium surface (Fig. 2d(a1)). These were produced during the grinding process. At high resolution, the surface of uncoated titanium was smoothed (Fig. 2d(a2)). Figure 2d(b1,b2) show the granular-like structures that appeared on the titanium surface after the assembly of the CS-(HA-LDc)5 multilayer. At high resolution, island-like structures were observed on surfaces with multilayer coatings. This difference in surface morphology can primarily be attributed to crosslinking.

### *In-vitro* gene transfection

**Figure 3:**
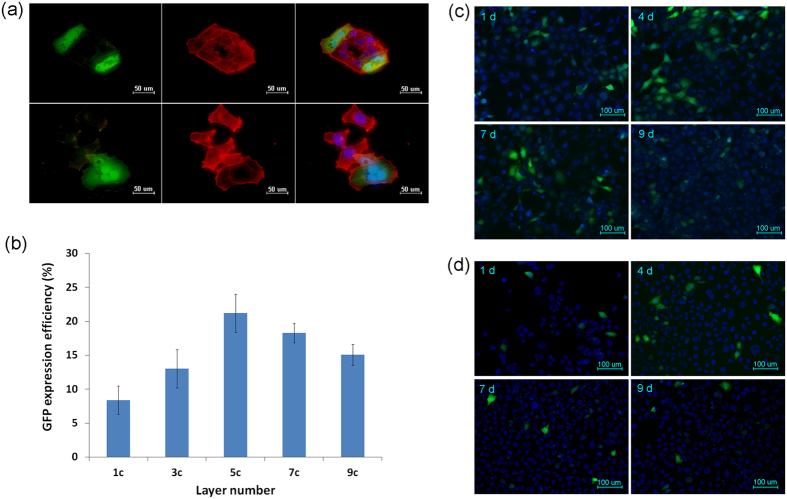
*In vitro* gene transfection evaluation. **(a)** GFP expression in HEK293 cells: green fluorescence (GFP); red fluorescence (cytoskeleton); blue fluorescence (nuclear), under ×400 magnification. **(b)** GFP expression efficiency of HEK293 cultured on CS-(HA-LDc)n. **(c)** GFP expression of HEK293 cultured on CS-(HA-LDc)5 after culturing for 1 d, 4 d, 7 d and 9 d, under ×200 magnification. **(d)** GFP expression of HN4 cultured on CS-(HA-LDc)5 after culturing for 1 d, 4 d, 7 d and 9 d, under ×200 magnification.

As shown in Fig. 3a, GFP expression in HEK293 cells was evaluated. As the number of layers increased, up to five, the efficiency of GFP expression also increased. However, as the number increased beyond five layers, the efficiency of GFP expression instead decreased (Fig. 3b). Expression of GFP reached peak efficiency when the assembly had five layers. Results showed that the expression of GFP could last for longer than one week (Fig. 3c). The efficiency of GFP expression in HEK293 cells on the surface of CS-(HA-LDc)5 layers was highest (20.36 ± 7.25%) after 4 days of culture, and the efficiency of GFP expression decreased to 12.51 ± 6.33% after 7 days of culture. In Fig. 3d, it shows representative results of HN4 cell transfection after 1 day, 4 days, 7 days, and 9 days of culture. Similarly, the efficiency of GFP expression was highest (12.38 ± 3.63%) at 4 days.

### Cell adhesion in early stages

**Figure 4:**
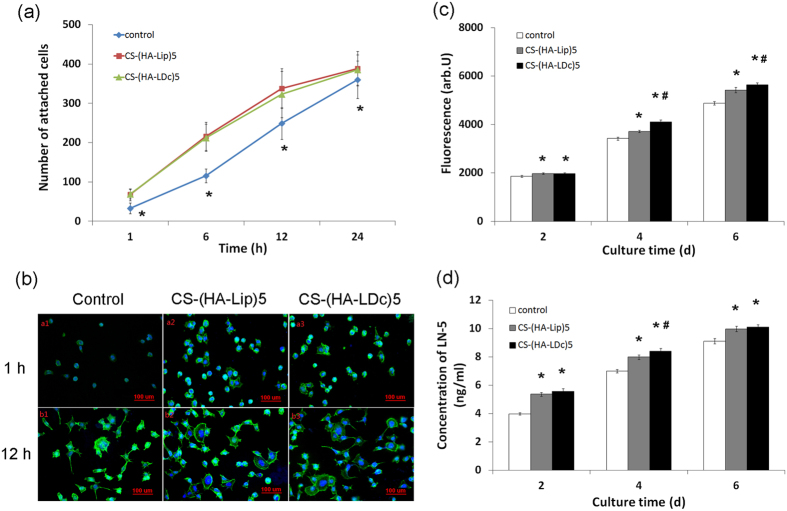
Biological evaluation of HEK293 cells on different surfaces. **(a)** Cell adhesion assay of early stage. Number of HEK293 cells attached to substrata (control, CS-(HA-Lip)5, CS-(HA-LDc)5 after 1 h, 6 h, 12 h and 24 h of culture. Ten randomly chosen fields (1.2 mm^2^) per sample were evaluated. The values = means ± SD. Statistical analysis by ANOVA: *P < 0.05 versus CS-(HA-Lip)5 and CS-(HA-LDc)5 at the corresponding time point. **(b)** The fluorescence microscopic photo showing morphology of HEK293 cells cultured for 1 h and 12 h on control (a1, b1), CS-(HA-Lip)5 (a2, b2), CS-(HA-LDc)5 (a3, b3). **(c)** Cell proliferation and attachment on different groups were determined by fluorescence assay. *Statistically significant difference between the CS-(HA-Lip)5 or CS-(HA-LDc)5 group and the control group (*P* < 0.01); ^#^Statistically significant difference between the CS-(HA-Lip)5 and CS-(HA-LDc)5 group (*P* < 0.01). The data shown are the mean fluorescence value ± SD (n = 3). **(d)** Quantification of Laminin-5 secretion in HEK293 cells culture on different surfaces. *Statistically significant difference between the CS-(HA-Lip)5 or CS-(HA-LDc)5 group and the control group (*P* < 0.01); ^#^Statistically significant difference between the CS-(HA-Lip)5 and CS-(HA-LDc)5 group (*P* < 0.05). The data shown are the mean concentration value ± SD (n = 3).

The number of attached cells was determined to evaluate HEK293 cell adhesion. Cells attached to titanium were observed using a fluorescence microscope. From 1 to 24 h after cell seeding, significantly more attached cells were seen on coated surfaces than on the control surface (Fig. 4a). However, there was little change in the number of attached cells on CS-(HA-Lip)5 or on CS-(HA-LDc)5 from 1 h to 24 h after plating. At 1 h, polygonal cells with pseudopodia were observed on coated surfaces, while on the control surface, most cells had not spread and remained round in shape. Most cells on the three substrates had spread after 12 h of culture, but the average area of HEK293 cells was smaller on the uncoated titanium surface than on the coated titanium surface. The area of HEK293 cells was not significantly different between CS-(HA-Lip)5 and CS-(HA-LDc)5 (Fig. 4b).

### Cell proliferation and adhesion

To characterize the effects of multilayered DNA coatings that might affect cell viability, the Alamarblue assay was used to determine the number of HEK293 cells on each surface after 2 days, 4 days, and 6 days. As shown in Fig. 4c, both the CS-(HA-LDc)5 and CS-(HA-Lip)5 displayed significantly higher fluorescence, indicating that there were more cells on these surfaces than on uncoated titanium after 4 days and 6 days of culture (*P* < 0.01). Moreover, there were significantly more cells on the surface of CS-(HA-LDc)5 than on that of CS-(HA-Lip)5 layers after 4 days of culture (*P* < 0.01). These results indicated that the coatings fabricated using the layer-by-layer assembly technique improved the cytocompatibility of the native titanium surface.

### Quantification of laminin-5

As shown in Fig. 4d, the concentration of laminin-5 secreted by HEK293 was significantly different on the three surfaces after 4 days of culture (*P* < 0.01). Both the CS-(HA-LDc)5 and CS-(HA-Lip)5 displayed significantly higher concentrations of laminin-5 than the control group after 2 days and 6 days of culture (*P* < 0.05). However, there was no significant difference in the concentration of laminin-5 between the surfaces of titanium coated with CS-(HA-LDc)5 and CS-(HA-Lip)5 (*P* > 0.05).

### Immunofluorescence staining of laminin γ2 and hemidesmosomal components

**Figure 5:**
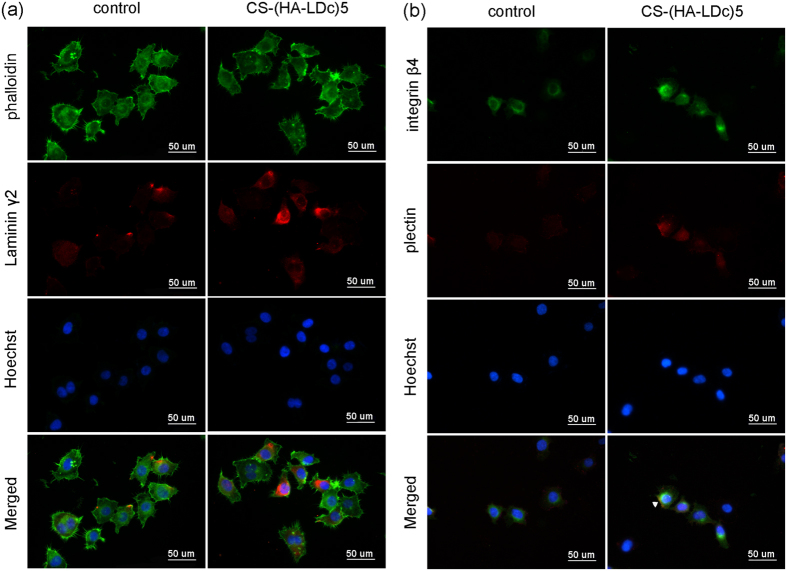
Effects of CS-(HA-LDc)5 coating on the expression of laminin γ2 and hemidesmosomal components in HN4 cells. **(a)** Immunofluorescent microscopy expression of laminin γ2 in HN4 cells (red); cytoskeleton (green); nuclei (blue). **(b)** Integrin β4 was in green, plectin was in red and nuclei were in blue. Co-localized staining in the merged image appeared in yellow, indicating the HD-like structure (white arrow head). Samples were examined under ×400 magnification.

The cytoskeleton was labeled with green fluorescence, laminin γ2 was detected as red fluorescence, and nuclei indicated by Hoechst stains were observed as round areas of blue fluorescence (Fig. 5a). Weak immunoreactivity of laminin γ2 was observed in the cytoplasm of HN4 cells in the control group, while the expression of laminin γ2 was quite obvious in several HN4 cells cultured on the CS-(HA-LDc)5 coating. This demonstrated that HN4 cells expressed laminin γ2 without transfection, and that the CS-(HA-LDc)5 coating could enhance the expression of laminin γ2. In Fig. 5b, integrin β4 is indicated in green, plectin in red, and nuclei in blue. Faint immunoreactivities for integrin β4 and plectin were observed in the cytoplasm of HN4 cells in the control group. In the transfected group, the co-localization of integrin β4 and plectin was observed on one side of a HN4 cell; this indicated the formation of hemidesmosomes on the CS-(HA-LDc)5 coating.

## Discussion

Laminin-5 is an epithelium-specific adhesion-related protein, and it contains a unique γ2 chain that can promote epithelial sheet migration over a wound bed^25^,^26^. A previous study demonstrated that titanium plates modified with multilayer DNA coating assembled using the layer-by-layer technique could transfer plasmid DNA into cells and subsequently influence cell function^24^. In this study, the surface of polished titanium disks was modified using the layer-by-layer technique with CS, HA, and cationic lipid–DNA (laminin γ2) complexes, using methods described in previous reports^23^,^24^. The results supported the conclusion that the gene coatings were successfully assembled on the titanium surface. The final *in-vitro* results demonstrated that this delivery system was able to control the release of plasmid DNA and that this layer-by-layer coating enhanced cell adhesion.

Fluorescence microscopy of labeled DNA-loaded titanium surfaces directly indicated that the layer-by-layer assembly process produced surfaces coated with laminin γ2 cDNA, which increased in DNA content with increasing number of bilayers. Wide X-ray photoelectron spectroscopy revealed that the titanium surfaces after assembly consisted of Ti, O, C, N, Na, and P, and that the concentration profile of P revealed the presence of cDNA (laminin γ2). The significant increase in the intensity of N on the layer-by-layer films may result from the fabrication of DNA and HA. The disappearance of the Ti(2p3/2) and Ti(2p1/2) peaks suggest that the area of coatings covering the titanium surface increased in size and that adsorbed polyelectrolytes formed a thin film on the titanium surfaces. The results of this study show that the coatings were successfully established on the surface of the titanium. Changes in the surface topography and the increase in hydrophilicity also indicated that the coatings were assembled onto the titanium surface in turn.

In such a coating, the multilayers are formed by electrostatic attraction between oppositely charged constituents. Chitosan is colloidal in weak acid medium with a positive charge. Hyaluronic acid is a major component of the extracellular matrix and carries a negative charge. In this way, tailoring the polyelectrolyte of the polysaccharide CS by crosslinking to HA resulted in a long degradation period^27^. The degradation behavior of the crosslinked coating in PBS solutions indicated their ability to transfer laminin γ2 locally into cells. Similar results have been reported by other researchers^24^,^28^. A previous study reported that the transfection efficiency of cells gradually increased as the number of layers increased^23^. However, the results of this study showed that the GFP expression efficiency was highest when there were five layers in the assembly. It is here reported that cationic liposomes are adsorbed by negative charges on the surface of the cell membrane and carried into cells through endocytosis. In this way, cationic liposomes with excess positive charge are critical to transfection efficiency; the transfection efficiency is higher when cationic liposomes carry more positive charge^29^,^30^. However, by increasing the number of layers, the negative charge carried by HA also increased, which allowed HA to compete with the negative charge on the surface of the cell membrane. This may explain why the efficiency of GFP expression efficiency decreased when there were more than five layers. For this reason, HEK293 cells were incubated directly on multilayer CS-(HA-LDc)5 titanium surfaces, while cells incubated on multilayer CS-(HA-Lip)5 titanium surfaces served as negative controls. In this study, the expression of GFP remained observable for 9 days, a finding consistent with results indicating the release of plasmid DNA. However, over time, as proliferation and cellular apoptosis continued, the number of transfected cells declined because the cells had been transiently transfected.

The current study of initial attached cell number and morphology indicated that both coatings strengthened the initial attachments made by HEK293 cells. Previous reports have suggested that cell attachment to substrates is influenced by the balance of hydrophilicity and hydrophobicity and the presence of adhesion molecules^31^. In this study, the cells transfected with the plasmids were unable to express laminin γ2 until 24 h after layer-by-layer assembly, while the surface of titanium immediately became more hydrophilic after layer-by-layer coating with CS-(HA-LDc)5 or CS-(HA-Lip)5. It is therefore likely that the initial number of attached cells was influenced primarily by the hydrophilic coating on the surface because of the hydrophilic nature of HA and DNA. These results were consistent with the results of previous reports, which suggested that the higher the hydrophilicity of a material surface, the stronger attachment of the cells to the material^32^. In this study, immediately after plating, HEK293 cells were round in shape and then spread to become flat. The rapid increase in cell area on coated titanium disks after 1 h and 12 h suggested that cells on coated titanium disks began to spread earlier than cells on uncoated titanium disks. Furthermore, initial spreading is an important factor affecting initial proliferation^31^. After 2 days, cells cultured on the assembled surface showed more cell proliferation than those on the uncoated surface. As expected, cell attachment and proliferation on CS-(HA-LD)5-coated surfaces were better than on CS-(HA-Lip)5-coated and uncoated surfaces. It was therefore concluded that laminin γ2 gene coatings on the titanium surface could improve overall cell attachment and proliferation. However, the proteolytic processing of laminin γ2 is also bound up with keratinocytes to switch from an adhesive to a migratory state^33^. It is unclear how laminin-5 regulates the apparently opposite cellular functions of cell adhesion and cell migration. In addition, the transfection efficiency and the capacity of coating titanium surfaces with multilayer laminin γ2 DNA coatings must be improved.

HEK293 cells regulate expression of functional laminin-5 molecules via transfection of laminin γ2. The current results indicate that significantly more laminin-5 had been deposited by transfected cells than by the other two groups after 4 days culture. However, the difference was not significant on either day 2 or day 6. This is consistent with the results showing the percentage of transfected cells, which was determined using GFP. Owing to cell proliferation and transient transfection, the concentration of recombinant protein became lower as the culture progressed. In transient gene expression, the gene encoding the protein of interest is not integrated into the host genome and the exogenous gene is only expressed for a short period of time before the culture is terminated^17^. As proliferation and cellular apoptosis continue, the number of transfected cells declines, resulting in a decline in secretion of the laminin γ2 chain. The difference between CS-(HA-Lip)5 and uncoated titanium may also contribute to the function of HA, which could regulate cell proliferation. These results confirm previous observations showing that the γ2 chain is required for deposition and incorporation of laminin-5 into the extracellular matrix, which may be relevant to the proteolytic processing of the γ2 chain from 150 kD to 105 kD^12^. However, the mechanism underlying proteolytic processing of the laminin γ2 chain in the formation of the epithelial basement membranes remains unclear.

The expression of laminin γ2 in HN4 cells was more intense and diffusely distributed over the cytoplasm when cultured on the CS-(HA-LD)5-coated surfaces, at least as assessed by immunofluorescence. These results indicated that the multilayer laminin γ2 DNA coating could promote the expression of laminin-5 in epithelial cells, which was beneficial for the cell adhesion associated with integrin α6β4 at hemidesmosomes. Additionally, the cytoplasmic domain of integrin β4 is responsible for interactions with the three major hemidesmosomal components plectin, BP180, and BP230^34^. In this study, integrin β4 and plectin were immunolabeled by double color immunofluorescence to locate the hemidesmosomes. As shown in the merged image of HN4 cells cultured on the CS-(HA-LD)5 coating, co-localized staining appeared in yellow was observed, which indicated the hemidesmosome-like structure. The multilayer laminin γ2 DNA coating could promote the formation of hemidesmosomes in epithelia cells, which was beneficial in strengthening the attachment of the epithelium to the surface of implant.

In conclusion, a multilayer structure consisting of cationic lipid and laminin γ2 DNA can be fabricated on titanium using the layer-by-layer assembly process. The DNA-loaded multilayer provides the surface with good biocompatibility. Plasmid DNA was efficiently transferred to HEK293 cells cultured on the multilayer substrate surface, which showed a better attachment and proliferation ability than cells cultured on uncoated surfaces. The expression of laminin γ2 and the co-localization of integrin β4 and plectin were more obvious in HN4 cells cultured on the multilayer laminin γ2 DNA coating. The DNA-loaded multilayer coating was here found to be a convenient and effective means of improving cell adhesion and the formation of hemidesmosomes on titanium surface.

## Additional Information

**How to cite this article**: Yang, G. *et al.* Fabrication, characterization, and biological assessment of multilayer Laminin γ2 DNA coatings on titanium surface. *Sci. Rep.* **6**, 23423; doi: 10.1038/srep23423 (2016).
